# Evaluating the Implementation of a Twitter-Based Foodborne Illness Reporting Tool in the City of St. Louis Department of Health

**DOI:** 10.3390/ijerph15050833

**Published:** 2018-04-24

**Authors:** Jenine K. Harris, Leslie Hinyard, Kate Beatty, Jared B. Hawkins, Elaine O. Nsoesie, Raed Mansour, John S. Brownstein

**Affiliations:** 1 Brown School, Washington University in St. Louis, St. Louis, MO 63130, USA; 2 Center for Health Outcomes Research, Saint Louis University, St. Louis, MO 63104, USA; hinyardl@slu.edu; 3 Center for Interprofessional Education & Research, Saint Louis University, St. Louis, MO 63104, USA; 4 Health Services Management and Policy, College of Public Health, East Tennessee State University, Johnson City, TN 37614, USA; beattyk@mail.etsu.edu; 5 Computational Health Informatics Program, Boston Children’s Hospital, Boston, MA 02115, USA; Jared.Hawkins@childrens.harvard.edu (J.B.H.); John.Brownstein@childrens.harvard.edu (J.S.B.); 6 Department of Pediatrics, Harvard Medical School, Boston, MA 02115, USA; 7 Institute for Health Metrics and Evaluation, University of Washington, Seattle, WA 98121, USA; en22@uw.edu; 8 Chicago Department of Public Health, Chicago, IL 60604, USA; Raed.Mansour@cityofchicago.org

**Keywords:** food safety, implementation, local health department, Twitter, consolidated framework for implementation research, CFIR

## Abstract

Foodborne illness is a serious and preventable public health problem affecting 1 in 6 Americans with cost estimates over $50 billion annually. Local health departments license and inspect restaurants to ensure food safety and respond to reports of suspected foodborne illness. The City of St. Louis Department of Health adopted the *HealthMap Foodborne Dashboard* (Dashboard), a tool that monitors Twitter for tweets about food poisoning in a geographic area and allows the health department to respond. We evaluated the implementation by interviewing employees of the City of St. Louis Department of Health involved in food safety. We interviewed epidemiologists, environmental health specialists, health services specialists, food inspectors, and public information officers. Participants viewed engaging innovation participants and executing the innovation as challenges while they felt the Dashboard had relative advantage over existing reporting methods and was not complex once in place. This study is the first to examine practitioner perceptions of the implementation of a new technology in a local health department. Similar implementation projects should focus more on process by developing clear and comprehensive plans to educate and involve stakeholders prior to implementation.

## 1. Introduction

Foodborne illness is a serious and preventable public health problem affecting 1 in 6 Americans with estimated costs of more than $50 billion annually in the U.S. [1,2]. Few who become sick seek medical care and most do not report their illness [1,3]. As a result, existing surveillance systems only capture a small fraction of the disease burden, making development and evaluation of food safety policies and programs challenging [4,5].

Nationwide, local health departments (LHDs) license and inspect restaurants to ensure food safety. In addition to regular licensing and inspections, LHDs respond to reports of suspected foodborne illness submitted by consumers and healthcare providers. Although the relationship is complex [6], some evidence suggests restaurants with lower food safety scores during inspections may have higher foodborne illness outbreak rates [7]. In addition, inspections prompted by consumers identify more serious and critical violations than regular inspections, leading to a higher proportion of failed inspections [8]. To improve surveillance and reporting, two large LHDs have begun to incorporate new media into their food safety efforts. In 2012–2013, the New York City Department of Health and Mental Hygiene used *Yelp* restaurant reviews to identify restaurants where patrons became ill [8]. In 2013–2014, the Chicago Department of Public Health mined Twitter for food poisoning tweets with the web application FoodBorne Chicago [9]. New York and Chicago were successful in identifying and addressing serious and critical food safety violations based on consumer complaints through new media sources [8,9], providing evidence suggesting new media may be a useful tool to incorporate into food safety efforts.

To facilitate the incorporation of new media into food safety efforts, the Computational Epidemiology Group at Boston Children’s Hospital developed the *HealthMap Foodborne Dashboard* (Dashboard) [10]. Like FoodBorne Chicago, the Dashboard uses an algorithm to monitor Twitter for any tweets containing “food poisoning” or “foodpoisoning” in a geographic area. Relevant tweets that contain the key words and are in the geographic area are displayed in real-time in a list on a web-based interface. The list shows each tweet collected by the algorithm and has options allowing health department staff to reply to, discard, or view each tweet in more detail. Given the success of social media for foodborne illness reporting and surveillance in Chicago and New York, and evidence that social media use can improve the relationship between government and the public [11,12], we adopted, adapted, and implemented the HealthMap Dashboard in the City of St. Louis Department of Health (STL-DOH).

While the Dashboard had already been developed and its functionality has been tested, it had not yet been implemented and evaluated in a practice setting and the Chicago and New York projects did not report on the implementation process. To provide other practice settings with more information about the implementation process for a new tool like the Dashboard, we conducted semi-structured qualitative interviews with employees involved in food safety at the STL-DOH to evaluate the implementation [13]. This manuscript reports the results of these interviews and the implications for implementation of similar technologies in practice settings.

## 2. Materials and Methods

Before implementation of the Dashboard, there were seven methods available for consumers to report suspected foodborne illness in St. Louis: phone call, email, fax, completed web form, tweet sent to the City of St. Louis Citizens’ Service Bureau (@stlcsb; CSB), direct contact from the STL-DOH, or other direct contact [14]. When a complaint is received through any of these mechanisms, the CSB enters the complaint into a CityWorks database. Foodborne illness complaints are then routed by the CSB to the STL-DOH through a connection between CityWorks and the environmental health software HealthSpace. At the STL-DOH, complaints are accessed by the Food & Beverage Control division within the STL-DOH and assigned to an inspector.

In fall 2015, the Dashboard was adapted for use in St. Louis and added to the mechanisms for reporting foodborne illness to the STL-DOH [14]. Adaptation required selecting geographic coordinates as parameters for tweet collection; we set the boundary for tweet collection at a 50-mile radius around St. Louis. Adaptation also included modifying the format of information sent from the Dashboard to the STL-DOH when a report was filed so that it could be received by CSB with all the same information collected in other types of reports to enter into CityWorks just as any other type of report would be. Once in CityWorks, reports filed via the Dashboard were indistinguishable from reports from other mechanisms (e.g., phone). This project was named Food Safety STL.

### 2.1. Sample Selection

Following the implementation of the Dashboard, we approached STL-DOH personnel involved in the implementation including epidemiologists, environmental health specialists, health services specialists, food inspectors, CSB personnel, and public information officers. At least one person in each position agreed to be interviewed, with the exception of CSB. Written consent was provided by each participant in person before the interviews began. The Washington University in St. Louis Institutional Review Board approved the study (IRB ID # 201601097).

### 2.2. Interview Guide

We based the interview guide on the Consolidated Framework for Implementation Research (CFIR) [15]. CFIR has five domains important to effective implementation: (1) Intervention characteristics: key attributes of interventions that influence the success of implementation (e.g., the complexity of the intervention); (2) Outer setting: characteristics of participants and external partners and stakeholders that influence implementation success (e.g., the needs and resources of the participants); (3) Inner setting: characteristics of the intervention setting that influence implementation success (e.g., the culture of the organization); (4) Characteristics of individuals: the interaction between people, including stakeholders and participants, and the intervention (e.g., knowledge about the intervention); and (5) Process: aspects of planning, engaging, executing, and evaluation of the implementation that influence implementation success. Interview questions focused on three domains: intervention characteristics, outer setting, and inner setting (Table 1).

Interview responses were analyzed following the CFIR qualitative analysis plan [16]. Although we asked questions in just three of the five CFIR domains, we coded participant responses to any applicable CFIR domain and to the constructs and sub-constructs within the domain. Three coders (J.K.H., L.H., K.B.) coded each block of text in each interview to the domains, constructs, and sub-constructs. Following the CFIR guidelines, each response was coded to a construct as positive, negative, neutral, or mixed with respect to the implementation of Food Safety STL. Positive and negative codes were also coded as weak or strong. Six of the transcripts were coded by two coders, one was coded by all three coders. Coding teams met to discuss codes and came to consensus on the final set of codes for each of the transcripts. After coming to consensus, the three coders assigned 34 of the CFIR constructs or sub-constructs across all five CFIR domains. Constructs and sub-constructs coded five or fewer times overall after consensus were subsequently omitted from results reporting. De-identified data showing interview text and codes assigned are available in an online repository [17].

## 3. Results

The seven interview participants held the positions of epidemiologist, environmental health supervisor, two health services managers, two food establishment inspectors, and public information officer. The mean interview length was 35.6 min (s.d. = 12.1) with the shortest interview at 22.3 min and the longest interview lasting 56.3 min.

**Figure 1:**
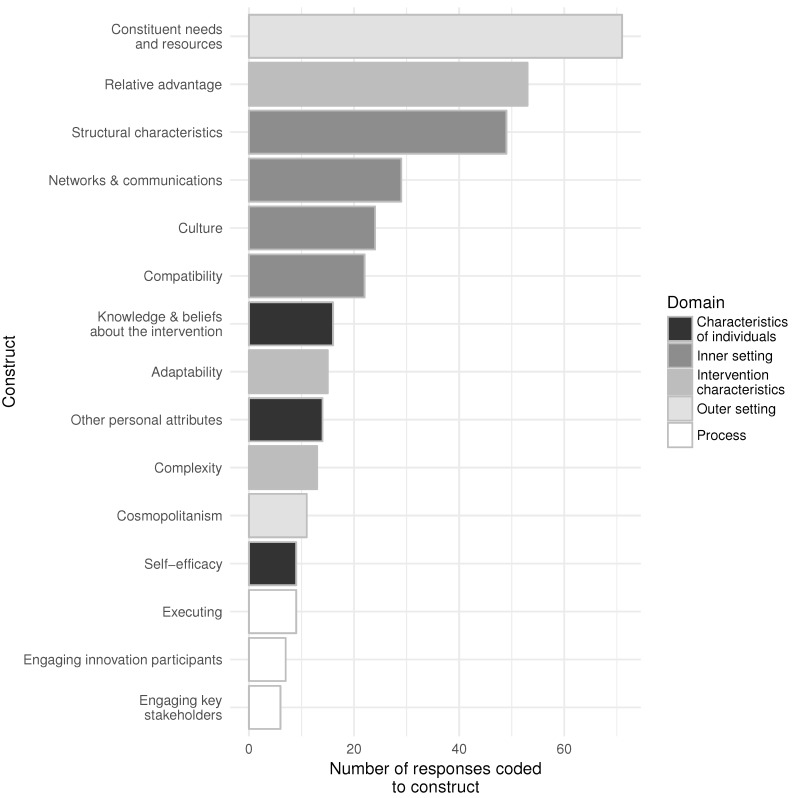
Domains and constructs represented in interviews of health department employees after Food Safety STL implementation.

Fifteen constructs in five domains were coded more than five times (Figure 1). The most commonly coded construct was constituent needs and resources from the outer setting domain, which was coded 71 times across the seven interview transcripts. The least commonly coded construct was engaging key stakeholders, which was coded six times. The three domains explicitly included in the interview guide were coded the most often with inner setting coded 124 times, outer setting coded 82 times, and intervention characteristics coded 81 times. Characteristics of individuals and process were coded 39 and 22 times, respectively. Statistical code used for all analyses and figures are available in an online repository [17].

**Figure 2:**
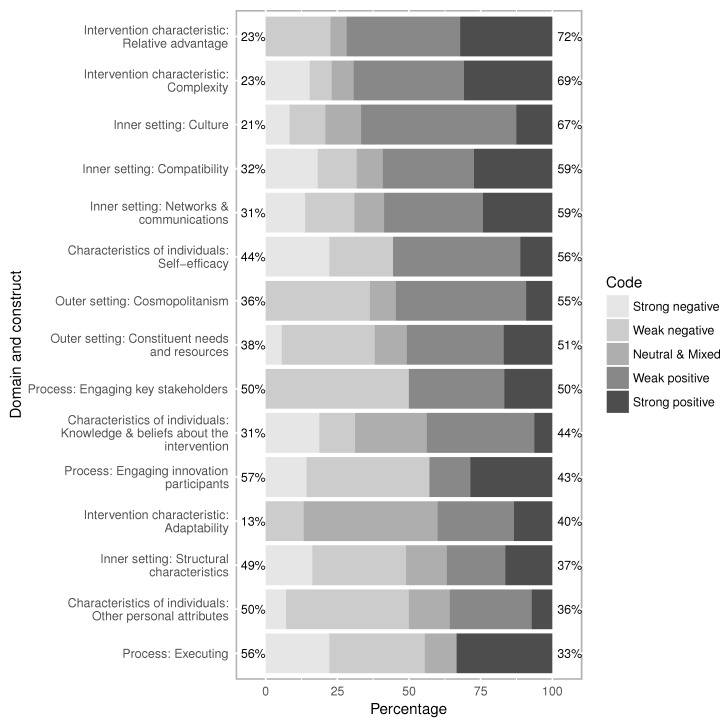
Percentage of ratings for all constructs with more than five mentions across all interviews.

All constructs were deemed strongly positive with respect to the implementation of Food Safety STL in at least one statement, while 11 of the 15 constructs were perceived as strongly negative in at least one statement (Figure 2). Four constructs were viewed more negatively than positively, one was equally negative and positive, and 10 were viewed more positively than negatively. Two of the three constructs from the process domain (engaging innovation participants, executing) were among the four viewed more negatively and the third process item, engaging key stakeholders, was viewed equally negatively and positively. Two intervention characteristics constructs, relative advantage and complexity, were viewed positively the most across all constructs, followed by three of the four inner setting constructs: culture, compatibility, and networks and communications. The adaptability construct was coded as neutral or mixed 47% of the time, higher than all other constructs by far.

**Figure 3:**
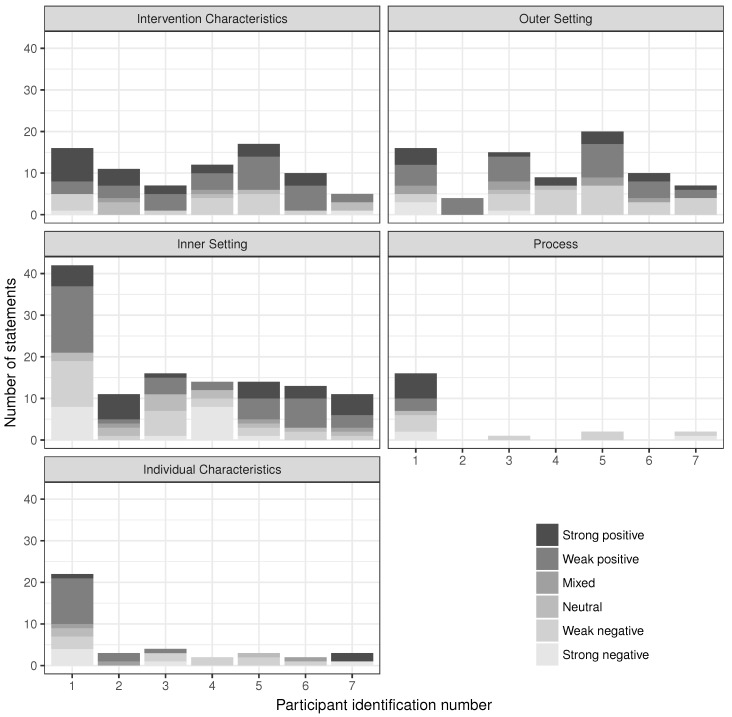
The number and tone of participant statements in each of the five domains.

All seven health department employees who participated in interviews contributed statements to each domain except process, where only four of the seven participants made any statements (Figure 3). Overall, participant 1 contributed the most statements (n = 112; 32.9%), while participant 2 contributed the fewest statements (n = 29; 8.5%). Participant 1 was more active than all other employees in three domains: inner setting, process, and individual characteristics. Participant 5 was the most active in the other two domains. All participants contributed positive and negative statements.

### 3.1. Intervention Characteristics

All seven participants discussed the intervention characteristics domain, focusing on three constructs: adaptability, complexity, and relative advantage. Six participants discussed adaptability, making between one and four statements about adaptability. All seven participants discussed complexity, making between one and three statements about complexity. All seven participants discussed relative advantage; one participant made a single statement while the rest made between five and 14 statements about relative advantage. Complexity and relative advantage were perceived as positive traits of Food Safety STL most often, while adaptability was perceived as mostly neutral (Figure 2). Most perceptions of complexity indicated the intervention was not difficult to implement. For example, one participant stated:

Honestly, I think its goal is really big, but honestly, you guys have narrowed it down to a pretty simple procedure. This is even easier than if they call and then I have to go through the interview process. This is easier than what we normally do. So as far as intricacy goes, I think it doesn’t have too many steps. It’s really pretty streamlined. I really do. And I think for people who are afraid to pick up the phone and call but are willing to text or follow the steps online it makes it easier for them.

While participants mentioned multiple advantages both internally (e.g., improving connections among departments) and externally, there were two relative advantages that came up multiple times. First, several participants thought the Dashboard may be reaching St. Louisans who had not had much contact with the health department before:

Probably because we’re interested in reaching the public, and this is a way to capture another segment of the population that we might not capture. That’s the medium that they use, so that’s the medium that we want to get out there in.

Second, a few participants mentioned the pro-active strategy of reaching out to constituents, which demonstrates that the health department is actively working to protect the health of its constituents:

We were knocking on their doors to say, “Hey this blood lead level indicates that you have … your child has been exposed to lead in the last 30 to 60 days.” I think in those cases people were really quite happy to have the outreach and to learn about the services that were available in the city to address that. I think this could be very similar in that reaching out to somebody who complained of a foodborne illness that the city is proactive and that we want to address any issues that were coming up that we don’t have to just wait for you to come to us with a complaint.

Participants were unsure of the adaptability of the Dashboard; most suggested adapting the Dashboard to capture information from other social media platforms and using it to educate local constituents about food safety.

*I think you should involve other social media platforms or links, if there aren’t already. So I think it’s great that we’re giving people a channel to say, Hey, fill out this form. We’ll follow up with you. I think that shows a greater presence in the community that we’re reaching out to people, but I also think there is ... there’s always an opportunity for health promotion and education. So if there were a way for us to ... and I don’t know if you guys do this now … but to send out educational Tweets and so to kind of get a discussion about foodborne illness.*

Concerns regarding the adaptability, complexity, and relative advantage of the Dashboard included not believing people in St. Louis use social media in general or Twitter in particular, problems getting people to fill out the online form, and perceptions about harm to the food and beverage providers.

*There is this idea that people don’t use social media. So one of the inspectors told me, “Nobody uses Twitter. Why are you doing this project? Nobody uses Twitter.”*

*I think the only disadvantage at this point is that perception that it could potentially be harmful to food and beverage control providers … food and beverage providers in the city. But I think we’re addressing that hurdle.*

### 3.2. Outer Setting

All seven participants discussed the outer setting as well, focusing on two constructs, constituent needs and resources and cosmopolitanism. The constituents needs and resources construct measures to what extent the health department knows and prioritizes the needs of its constituents, while cosmopolitanism refers to how much the health department is connected with other organizations. While the majority of statements were positive, there were a substantial number of concerns as well.

Most of the positive perceptions of how Food Safety STL meets constituent needs and resources had to do with the reaching people where they are and increasing awareness of the health department through the pro-active strategy.

*I think it’s important that our residents feel like they can connect with us. I think a lot of the younger residents, they live on Twitter and social media, my generation, I guess, and so I think it allows a greater depth of communication. Yes, it’s just a Tweet, but that’s just another presence of the Health Department. In terms of people who would prefer more face-to-face contact, I think they still get that because there’s an inspector who’s going to come out and going to take a look, or we’re going to call you up, and so you still have that personal interaction, and so you feel like, hey, the Health Department cares.*

A few themes emerged in the negative perceptions of meeting constituent needs and resources. One common perception was that Food Safety STL does not meet the needs of populations without access to technology or who are hesitant to provide personal information via and online form due to a lack of trust:

I think that some of the barriers is that they receive ... say they receive a tweet from us asking them to report their illness. A hesitancy for not submitting that form or interacting is questioning the credibility of our handle. I think that’s probably one of the main things, and hesitancy with submitting their personal information.

Some participants were concerned that Food Safety STL would increase reports that were submitted in retaliation against a restaurant, for example:

Another disadvantage that it has compared to existing practices is that it’s also ... that we might also pick up on complaints that are defamatory or people who are tweeting about being sick but then putting another restaurant down. And I think that is a concern.

Several participants mentioned cosmopolitanism, or the extent to which the STL-DOH is linked to other organizations. Specifically, participants discussed how collaborations with local universities and neighboring health departments are improving the ability of STL-DOH to reach constituents and to evaluate programs. However, some also felt that Food Safety STL could create tension with food and beverage providers in the region and that the health department could build partnerships with local healthcare providers in order to improve food safety:

So there’s sort of a perception in the administration that this may target food and beverage providers in the city and that that may create some anxiety on their part and some tension between the public administration and the private citizens and businesses in the city.

It would also be nice if we could somehow get the actual doctors and clinics involved, because they don’t educate. They don’t ask for a stool sample. They don’t ask for a blood sample. They just go with self-defeating, “Drink some water, you’ll be better in 24 h.”

### 3.3. Inner Setting

All participants discussed the inner setting including STL-DOH culture, compatibility of the intervention with the STL-DOH, networks and communication, and structural characteristics. While culture, compatibility, and networks and communication were all perceived positively, there were more negative than positive comments about STL-DOH structural characteristics.

Positive perceptions of Dashboard compatibility, or how the intervention fits with existing workflows and systems, focused on how the maturity of the department facilitates innovation. A few participants mentioned that there was no change in workflow for most staff, while others were concerned that the implementation would result in a notable increase in submitted reports.

*The only thing that anybody had thoughts of was, oh my God, does this mean that we’re going to have a 50 percent increase in the number of complaints that we get? And people were freaking out for a very short second about that, but then when they realized that it’s not going to ... it’s not going to create that much more work, and actually we want that work because if people are sick, we need to know about it. Everybody calmed down; they just went about their business as usual.*

Most participants described the STL-DOH as having adequate or good networks and communication (formal and informal communication in an organization).

*The size of the department means that communicable disease and food and beverage control and epidemiology can work easily together because we know each other and we’re in close contact.*

### 3.4. Characteristics of Individuals

Three constructs related to the characteristics of individuals were mentioned more than five times. The knowledge and beliefs about the intervention construct is the familiarity of an individual with the intervention and their attitude toward it. The self-efficacy construct is belief in themselves to execute action to reach implementation goals. The other personal attributes construct captures traits that might influence the relationship of an individual to the intervention like motivation, values, competence, and learning style. Overall, knowledge about the intervention and self-efficacy were discussed more positively while personal attributes were more negatively than positively related to Dashboard implementation.

Most of the negative statements regarding personal attributes and self-efficacy were related to staff who struggle to use technology including computers in general and social media in particular.

*Well I’m sure that people that just are reluctant to use any kinds of social media that are not technology savvy, and that population is just ... whether it’s age or just fear of the medium ... I don’t know what we could do to change that type of ... that part of the culture of the organization because they don’t use it personally and...*

Statements regarding knowledge and beliefs about the intervention primarily described fascination and enthusiasm among staff tempered by some fear that the Dashboard would result in a lot of additional work.

*I think all of us food inspectors are fascinated by the idea. There were also some that were terrified that we would receive a whole bunch more bogus complaints, but that didn’t happen and that’s a good thing.*

### 3.5. Process

While the interview guide did not include questions on process, process constructs were mentioned by six of the seven participants and three process constructs were mentioned more than five times: engaging intervention participants, engaging key stakeholders, and executing. Engaging intervention participants’ statements refer to how participants engaged with the Dashboard and statements about engaging key stakeholders refer to how the Dashboard team and others at the health department interacted with the intervention. Executing statements are about carrying out the implementation according to plan. Process was viewed negatively at least as often as positively for all three process constructs.

Negative comments related to engaging intervention participants and key stakeholders indicated that both participants and key stakeholders are not familiar with Food Safety STL and hesitate to engage with it because they do not trust or do not understand the technology.

*I remember a couple months ago when the press release initially went to the mayor’s office, the first thing that they asked was like, “So now you’re asking citizens to tweet to you?” And I said, “No, no, no. We don’t want citizens to tweet to Food Safety STL.”*

*There is a hesitancy to initially report or submit that report form. And I think that citizens are still a little bit leery of the project, but I think as we continue to use it they are aware that the department is using alternative ways of surveillance to identify food-borne illness or outbreaks.*

In terms of executing the Dashboard, positive comments indicated that the Dashboard project has brought Divisions within the health department together to collaborate. Negative statements revolved mostly around the lack of buy-in from other government offices in the city.

*I think one barrier is that right now that we don’t have ... there’s yet to be a press release that has gone out, and if someone, whenever they get the questionnaire, at the same time, there could be a link that they could click on that would take them to an official press release that’s gone out or something that takes them to a city website that says, This is approved, this is legitimate, that would probably make the project more successful.*

## 4. Discussion

We interviewed STL-DOH staff about the implementation of the Food Safety STL Dashboard as a way to improve foodborne illness reporting in St. Louis. Constructs related to intervention characteristics and inner setting were viewed the most positively by participants while process was viewed the most negatively, suggesting one strategy for the Dashboard team in future implementations of the Dashboard is to develop more explicit plans to engage key stakeholders.

Several themes emerged during the interviews. The use of social media was seen as both a strength and a weakness across multiple domains. Some participants reported not understanding or personally struggling with new technology, not believing it is widely used, and feeling that it would not reach populations who do not have access to technology. Other participants described their colleagues who struggle with technology as older employees. Although we did not collect participant age, this perception is consistent with the lower levels of social media use by older adults [18] and the aging of the public health workforce [19]. Additional concerns about lack of support of external organizations, including other city government agencies and the restaurant association, also described a possible lack of understanding of social media and how the Dashboard works. Other health departments seeking to implement the Dashboard or similar technology should develop explicit plans for engaging and educating key stakeholders about the technology including health department professionals, members of local government outside the health department (e.g., City Mayor), and community organizations like the local restaurant association.

Some participants felt that St. Louisans might not trust outreach through social media channels. However, prior research has demonstrated that government use of social media can increase trust and improve perceived transparency [11], resulting in more interaction between the public and government. Notably, Chicago implemented a similar intervention and had much greater response from constituents [9,14]. In addition to being a larger city, Chicago has a very active civic social media presence through the Smart Chicago Collaborative and Open City, which use open data from the City of Chicago and other sources in a set of proprietary civic web apps. These apps encourage Chicagoans to connect with city data to do things like track crime in their neighborhood, find free condoms, and book a parking spot in advance. In comparison, the City of St. Louis has a very limited web and social media presence, which may result in some skepticism among residents who have never used civically-focused social media before.

Other participants felt the strategy was pro-active, could reach people through a channel they already use, and might facilitate interaction with St. Louisans who are not aware of, or connected to, their local health department. This sentiment is consistent with evidence that the interactive and real-time qualities of social media can be beneficial for communication between government and the public during emergencies and for everyday concerns [20]. Concerns about technology are likely to wane as the public health workforce, and the population in general, transitions from being comprised primarily of digital immigrants (i.e., people exposed to social media and other technologies later in life) to digital natives (i.e., people who grew up in the age of social media and other new technologies). Until this shift occurs, working to build trust and facilitate positive interaction via social media between the government and community members may require adoption of new tools for civic engagement overall (not just food safety) and public education on how to use these tools.

Participants thought the Dashboard could be improved by adapting it to capture other forms of social media and using it to aid in educating constituents by sending out food safety tweets on the @FoodSafetySTL Twitter feed. While Facebook is used far more widely than Twitter [18] and would be a logical addition to the Dashboard, Facebook does not currently make the needed data accessible so adding it to the Dashboard is not feasible. However, the Dashboard team is examining other data sources such as Yelp as possible additions to increase reach; Yelp reviews have been effective in identifying unreported foodborne illness in New York City [8]. One participant also mentioned the possibility of using the Dashboard data to improve surveillance, or the ability of the health department to understand and track foodborne illness in the jurisdiction, as well as reporting. Both Chicago and St. Louis are integrating complaints originating from a tweet captured by the Dashboard system into their foodborne illness surveillance systems [9,14]; other cities seeking to adopt the Dashboard could use it to enhance surveillance of foodborne illness, or, with a change of keywords, for surveillance of other public health concerns.

Several participants spoke of a tension between concerns that the Dashboard might increase workload immensely and the goal of improving food safety in the city by responding to constituent reports. Once the Dashboard was implemented, the volume of reports was relatively low and the increased workload did not emerge again as a concern, which was consistent with the experience of the Chicago Department of Public Health [9,14]. In addition, even if there were more incoming tweets, the increase to workload would not be projected to be large given the negligible time commitment per tweet.

This study was limited by possible self-report bias. Specifically, we asked participants to remember events that had occurred over several months; this can be associated with recall bias or selective memory bias. In addition, we were limited by the lack of a representative from the Citizens’ Service Bureau, which was one of the departments involved in the implementation process.

## 5. Conclusions

Strengths of the Dashboard implementation included easy integration into existing systems, no notable increase in workload, and the facilitation of pro-active communication with constituents. Challenges included concerns about greater workload and a lack of trust in, and capacity to use, social media. Despite its challenges and limitations, our study is the first to examine practitioner perceptions of the implementation of a new technology in a local health department. In light of our results, our primary recommendation for similar implementation projects is the development of a clear and comprehensive plan to educate and involve stakeholders about social media and the Dashboard prior to implementation.

## Figures and Tables

**Table 1:** Interview questions and Consolidated Framework for Implementation Research (CFIR) domains and constructs for evaluation of Dashboard implementation in the St. Louis Department of Health (STL-DOH).

Domain	Constructs	Interview Questions
*Intervention characteristics*	Relative advantage	How does Food Safety STL compare to other similar existing practices related to foodborne illness reporting at the DOH? What advantages does Food Safety STL have compared to existing practices? What disadvantages does Food Safety STL have compared to existing practices?
	Adaptability: the degree to which Food Safety STL could be adapted, tailored, refined, or reinvented to meet local needs	What kinds of changes or alterations do you think would be needed to make Food Safety STL work more effectively at the DOH? Do you think the DOH is capable of making these changes? Why or why not?
	Complexity: perceived difficulty of implementation of Food Safety STL	How complicated is Food Safety STL? Please consider the following aspects of Food Safety STL: duration, scope, intricacy and number of steps involved and whether Food Safety STL reflects a clear departure from previous practices.
*Outer setting*	Patient needs & resources	How well do you think Food Safety STL meets the needs of the individuals served by the DOH? In what ways did Food Safety STL meet their needs? E.g., convenient way of reporting? How do you think the citizens who used Food Safety STL responded to the service? What do you think are the barriers for the citizens participating or interacting with Food Safety STL?
*Inner setting*	Structural	How does the infrastructure of your organization (work hierarchy, age, maturity, size, or physical layout) affect the implementation of Food Safety STL? How does the infrastructure facilitate/hinder the implementation of Food Safety STL? How do you work around these structural challenges? What kinds of infrastructure changes are needed to further accommodate Food Safety STL? Changes in scope of practice? Changes in formal policies? Changes in information systems or electronic records systems? Other? What kind of approvals are needed? Who needs to be involved? Can you describe the process needed to make these changes?
	Culture: Norms, values, and basic assumptions of a given organization	How do you think your organization’s culture (general beliefs, values, assumptions that people have) affect the implementation of Food Safety STL? Can you describe an example that highlights this?
	Implementation climate	What is the general level of receptivity at the DOH to implementing Food Safety STL? Why?
